# Photoluminescence Properties of Layered Perovskite-Type Strontium Scandium Oxyfluoride Activated With Mn^4+^

**DOI:** 10.3389/fchem.2018.00467

**Published:** 2018-10-04

**Authors:** Hideki Kato, Yohei Takeda, Makoto Kobayashi, Hisayoshi Kobayashi, Masato Kakihana

**Affiliations:** ^1^Institute of Multidisciplinary Research for Advanced Materials, Tohoku University, Sendai, Japan; ^2^Department of Chemistry and Materials Technology, Graduate School of Science and Technology, Kyoto Institute of Technology, Kyoto, Japan

**Keywords:** photoluminescence, scandium oxyfluoride, layered perovskite, tetravalent manganese, red emission

## Abstract

In this research, we have found that layered perovskite titanate Sr_2_TiO_4_ doped with Mn^4+^ exhibits photoluminescence even at room temperature despite no luminescence from Mn^4+^-doped SrTiO_3_ with a three-dimensional bulky perovskite structure. The relative position of t_2g_ orbital of Mn to the valence band is a key factor for appearance of Mn^4+^-emission in Sr_2_TiO_4_:Mn. This result suggested usefulness of layered perovskite-type materials as hosts for Mn^4+^-activated phosphors than the bulky perovskite-type materials. Our investigation into photoluminescence of Mn^4+^-doped layered perovskite compounds has revealed that strontium scandium oxyfluoride Sr_2_ScO_3_F activated with Mn^4+^ exhibits Mn^4+^-emission with a peak at 697 nm under excitation at 300–600 nm and its emission intensity is much stronger than that of Sr_2_TiO_4_:Mn. The internal and external quantum yields of Sr_2_ScO_3_F:Mn were determined to be 50.5 and 43.5% under excitation at 345 nm, respectively.

## Introduction

**Figure 1:**
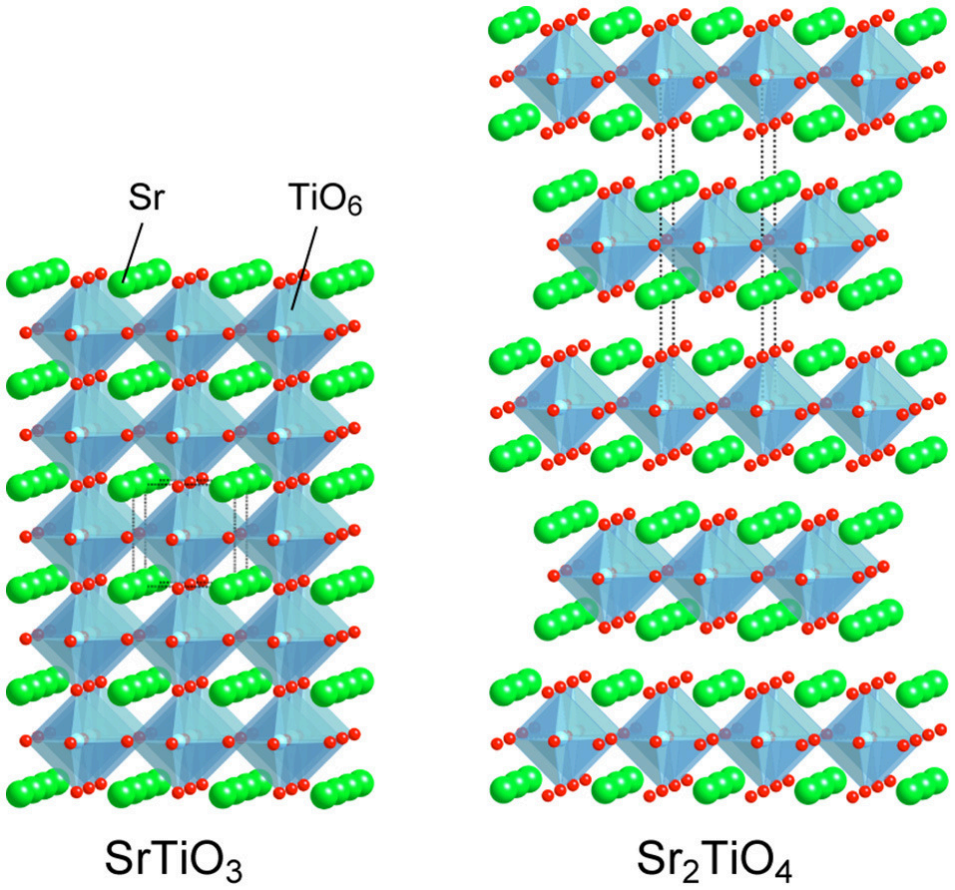
Crystal structures of SrTiO_3_ and Sr_2_TiO_4_.

White light emitting diodes (W-LEDs) based on blue-LEDs are widely spreading to various fields as highly efficient solid lightings (Lin et al., 2016; Adachi, 2018; Wang et al., 2018). Artificial white light is basically obtained by combination of blue and yellow light emitted from a blue-LED chip and a yellow-emitting phosphor Y_3_Al_5_O_12_:Ce, respectively. Such white light is inevitably cool white with high color temperature due to poor emission strength of Y_3_Al_5_O_12_:Ce in red region. Efficient red-emitting phosphors are added to achieve artificial warm white light by tuning color temperature. Nitride phosphors activated with Eu^2+^ such as (Sr,Ca)AlSiN_3_:Eu^2+^ and *M*_2_Si_5_N_8_:Eu^2+^ (*M* = Ca, Sr, and Ba) are extensively studied and commercially used as the red-emitting phosphors (Li et al., 2006, 2009; Uheda et al., 2006; Watanabe and Kijima, 2009; Tsai et al., 2015; Wang et al., 2018). However, requirements of high temperature and high pressure in synthesis of nitrides are drawbacks of nitride phosphors rising the costs. Therefore, development of alternative yellow—to red-emitting phosphors activated with Eu^2+^, which can be synthesized milder conditions in comparison with nitrides, is also conducted for oxides, phosphates, and oxyhalides (Toda et al., 2006; Daicho et al., 2012, 2018; Kim et al., 2013; Sato et al., 2014; Wen et al., 2016). Besides, phosphors activated with Mn^4+^ have been recently paid attention due to capability of red emission using wide variety of host materials (Srivastava and Beers, 1996; Seki et al., 2013; Ye et al., 2013; Sasaki et al., 2014; Wang et al., 2014; Takeda et al., 2015, 2017; Zhou et al., 2016; Cai et al., 2017; Wu et al., 2017; Xi et al., 2017; Zhang et al., 2017; Adachi, 2018; Jansen et al., 2018). Octahedral 6-fold coordination sites are preferred for substitution of Mn^4+^ ions. Fluorides and aluminates are paid much attention as hosts of Mn^4+^-activated phosphors from the viewpoints of their insulating nature and octahedral sites. Besides, titanates having semiconducting nature are also available for hosts of Mn^4+^-activated phosphors (Srivastava and Beers, 1996; Seki et al., 2013; Ye et al., 2013; Sasaki et al., 2014; Takeda et al., 2015; Zhang et al., 2017). We have recently reported that double perovskite-type titanates La_2_MTiO_6_ (M: Mg and Zn) are available as host materials of Mn^4+^-activated phosphors although a representative perovskite-type titanate SrTiO_3_ doped with Mn^4+^ could not show any luminescence at room temperature due to significant thermal quenching at low temperature, ~100 K (Takeda et al., 2015). Low temperature photoluminescence measurements and theoretical band structure calculations have revealed the importance of relative position of Mn 3d orbitals to valence and conduction bands of host materials in order to avoid electron transfer from the valence band to empty t_2g_ orbital of Mn and photoionization. The knowledge obtained from the previous researches encourages us to expand the research target for Mn^4+^-activated phosphors to Sr_2_TiO_4_ possessing a K_2_MgF_4_ type layered perovskite structure. Both SrTiO_3_ and Sr_2_TiO_4_ are members in a perovskite family composed of the same constituent elements. SrTiO_3_ of the representative perovskite-type compound is composed of TiO_6_ octahedra sharing corners infinitely, building three-dimensional bulky structure, while Sr_2_TiO_4_ has layers of the two-dimensional perovskite slab with a single TiO_6_ thickness separated by SrO layers as depicted in Figure 1. The decreases in structural dimension cause widening band gaps (Reyes-Lillo et al., 2016), which is thought to be a positive factor to suppress the electron transfer and/or the photoionization. Therefore, it is expected that comparison photoluminescence properties between SrTiO_3_:Mn and Sr_2_TiO_4_:Mn gives important information to understand the relationship between structural dimension and photoluminescence properties with Mn^4+^-activation.

In this research, we investigated photoluminescence properties of Mn^4+^-activated layered perovskite compounds. The differences in photoluminescence properties especially thermal quenching properties between SrTiO_3_:Mn and Sr_2_TiO_4_:Mn are discussed from features in crystal structures. In addition, we also investigated into photoluminescence properties of Sr_2_ScO_3_F:Mn possessing the K_2_MgF_4_ type structure as well as Sr_2_TiO_4_:Mn.

## Experiments

### Sample preparation

All powder samples were synthesized by a solid state reaction method using SrCO_3_ (Kanto, 99.9%), SrF_2_ (Wako, 99.5%), rutile type TiO_2_ (Kojundo Chemical, 99.9%), Sc_2_O_3_ (Shin-Etsu Chemical, 99.99%), and Mn(NO_3_)_2_ 6H_2_O (Wako, 98.0%) as raw materials. The stoichiometric mixtures of the raw materials were calcined at 1473 K for 5 h in air using alumina crucibles. Where, concentration of Mn substitution was fixed at 0.2 atom% to Ti or Sc. Non-doped samples were also synthesized by the same manner.

### Characterization of samples

Crystal phases of obtained samples were confirmed by powder X-ray diffraction (XRD) technique (Bruker, D2 Phaser). Photoluminescence measurements were performed using fluorescence spectrometers (Hitachi; F-4500 and Jasco; FP-6500). Photoluminescence spectra were also taken at low temperature (80–300 K with a step of 20 K) using a cryostat (Janis; VPF-475) under vacuum. Diffuse reflectance spectra of non-doped samples were taken by an absorption spectrometer equipping an integration sphere (Shimadzu; UV-3100). The band gaps of the non-doped samples with indirect transition were determined from (α*h*ν)^1/2^–*h*ν plot, where α, *h*, and ν represent Kubelka-Munk function, Planck constant, and frequency, respectively.

### Band structure calculation

The band structures were calculated by the plane wave based density functional theory (DFT) using CASTEP program (Payne et al., 1992; Milman et al., 2000). The Perdew-Burke-Ernzerhof (PBE) functional was used together with the ultrasoft-core potentials (Vanderbilt, 1991; Perdew et al., 1996, 1997). The cutoff energies were set to 300 eV. The electron configurations of the atoms were O: 2s^2^2p^4^, F: 2s^2^2p^5^, Sc: 3s^2^3p^6^3d^1^4s^2^, Ti: 3s^2^3p^6^3d^2^4s^2^, Mn: 3d^5^4s^2^, and Sr: 4s^2^4p^6^5s^2^. Super cells of Sr_16_Ti_7_MnO_32_ and Sr_16_Sc_7_MnO_25_F_7_ were employed for models of Sr_2_TiO_4_:Mn and Sr_2_ScO_3_F:Mn, respectively. Where, one F atom was also replaced with an O atom accompanied by the substitution of Mn for Sc to maintain the charge balance in the Sr_2_ScO_3_F:Mn system. From the experimental finding, the local electronic structure for the substituted Mn atom is known to be a 4+ cation, and the Mn ion is in the quintet state. Geometry optimization was carried out with respect to all atomic coordinates.

## Results and discussion

### Luminescence of Sr_2_TiO_4_:Mn

**Figure 2:**
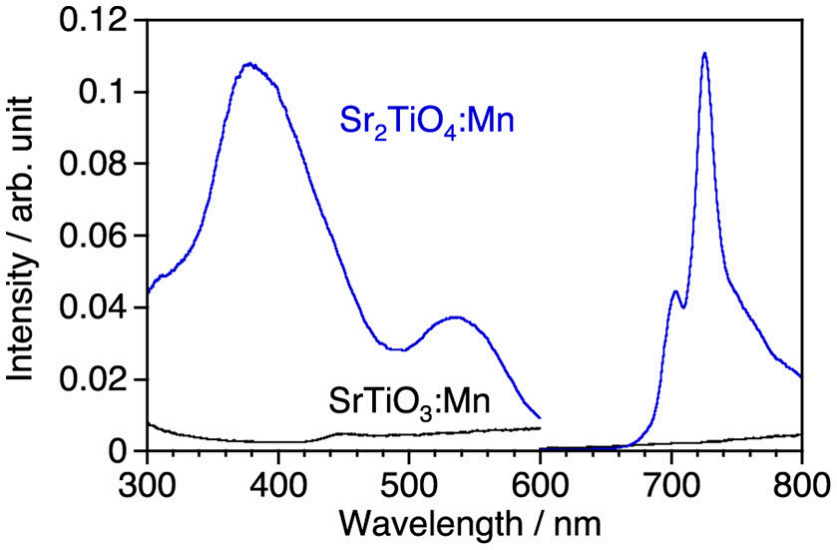
Luminescence spectra of SrTiO_3_:Mn and Sr_2_TiO_4_:Mn and corresponding excitation spectra at room temperature. Excitation and monitored wavelengths were 380 and 725 nm, respectively.

Figure 2 shows photoluminescence spectra of SrTiO_3_:Mn and Sr_2_TiO_4_:Mn with corresponding excitation spectra at room temperature. Sr_2_TiO_4_:Mn showed deep-red emission with a peak at 725 nm attributed to ^2^E_*g*_→^4^A_2g_ transition of Mn^4+^ under excitation at 300–580 nm. Although the emission intensity is not high, this is an interesting result taking into consideration of the fact that SrTiO_3_:Mn shows no emission at room temperature due to significant thermal quenching. Although both strontium titanates are composed of the same elements and are members of the perovskite family, a remarkable difference is present with regard to the structural dimension; Sr_2_TiO_4_ has a two-dimensional layered structure whereas SrTiO_3_ has a three-dimensional bulky one. Therefore, the appearance of Mn^4+^-emission in Sr_2_TiO_4_:Mn may reflect advantage of the layered perovskite structure in the band structure than bulky one. The further discussion about Sr_2_TiO_4_:Mn is described later.

### Comparison of luminescence properties between Sr_2_ScO_3_F:Mn and Sr_2_TiO_4_:Mn

**Figure 3:**
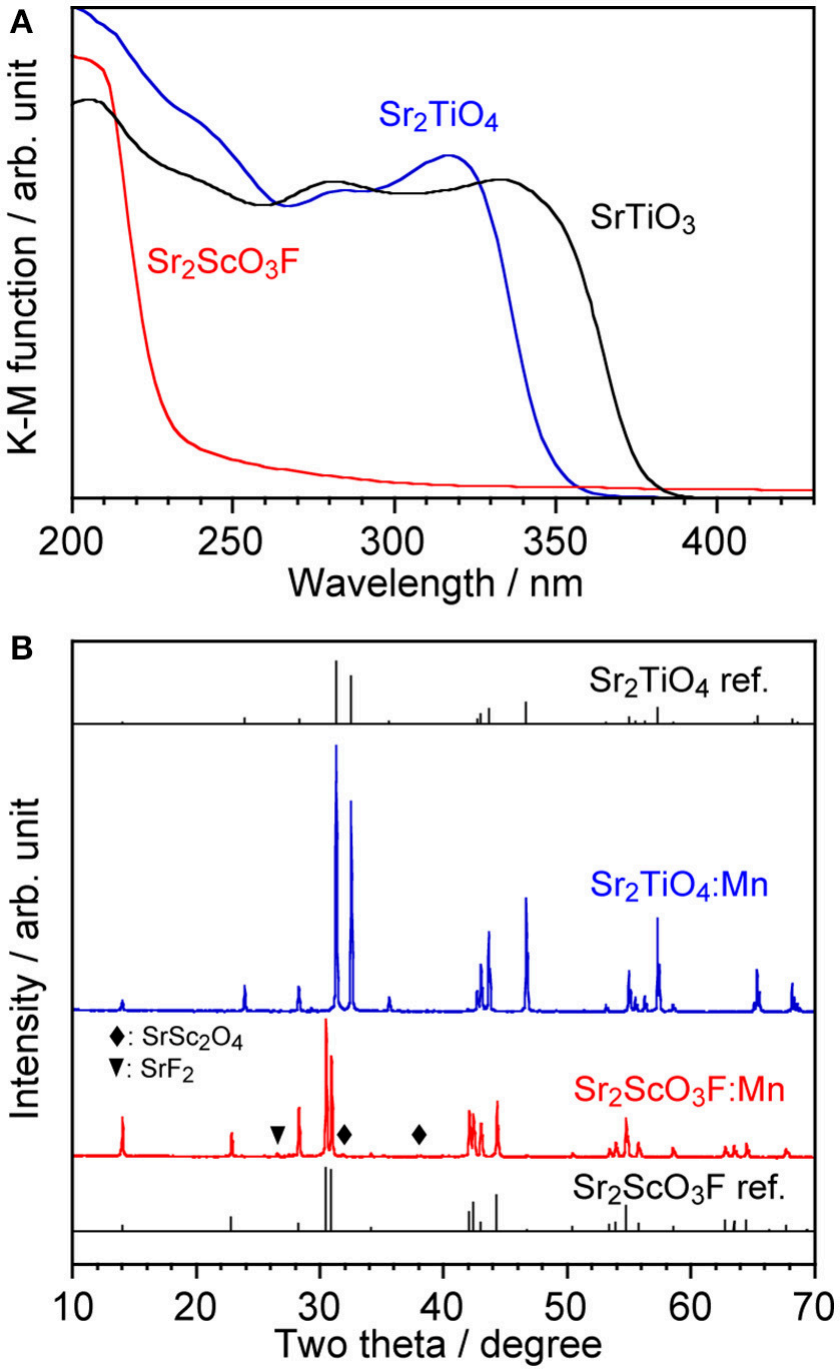
**(A)** Diffuse reflectance spectra of non-doped Sr_2_TiO_4_, Sr_2_ScO_3_F, and SrTiO_3_ and **(B)** XRD patterns of Sr_2_TiO_4_:Mn and Sr_2_ScO_3_F:Mn with reference patterns.

**Figure 4:**
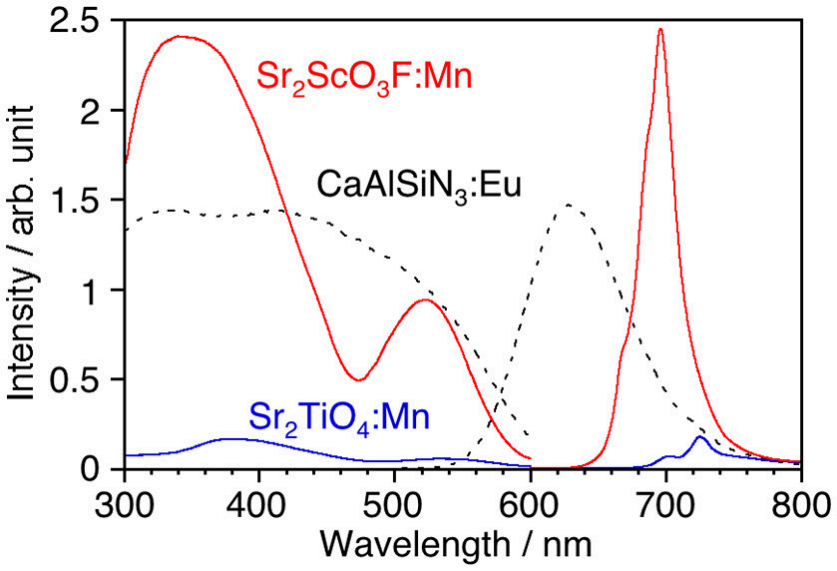
Luminescence spectra of Sr_2_TiO_4_:Mn, Sr_2_ScO_3_F:Mn, and CaAlSiN_3_:Eu with corresponding excitation spectra at room temperature. Excitation and monitored wavelengths were 380 and 725 nm for Sr_2_TiO_4_:Mn, 345 and 697 nm for Sr_2_ScO_3_F:Mn, and 340 and 630 nm for CaAlSiN_3_:Eu.

Although, as shown in Figure 3A, Sr_2_TiO_4_ has a wider band gap (3.46 eV) than SrTiO_3_ (3.21 eV) as reported in literature (Reyes-Lillo et al., 2016), other layered perovskite compounds possessing wider band gaps are preferred for efficient Mn^4+^-emission because of less probability of the electron transfer between Mn 3d and the valence and/or conduction band. Strontium scandium oxyfluoride Sr_2_ScO_3_F with a K_2_MgF_4_ type structure as well as Sr_2_TiO_4_, which has been recently discovered (Wang et al., 2015), was thought to be a good candidate because its octahedral building unit ScO_5_F based on the optically inert rare earth element was expected to give a wider energy gap in comparison with TiO_6_. XRD confirmed that Sr_2_ScO_3_F:Mn was obtained as the almost pure phase of Sr_2_ScO_3_F although it contained tiny amounts of SrF_2_ and SrSc_2_O_4_ as impurities whereas Sr_2_TiO_4_:Mn was obtained as a pure phase without any impurities (Figure 3B). Relative intensities of diffraction peaks of Sr_2_ScO_3_F:Mn at 14.1, 28.3, 43.0, and 58.5 degrees corresponding to reflections from (002), (004), (006), and (008), respectively, were remarkably strong in comparison with the standard ones due to orientation of crystals in (00*l*). The band gap of Sr_2_ScO_3_F has been discovered to be 5.38 eV, being wider than that of Sr_2_TiO_4_ (Figure 3A). Figure 4 shows emission and excitation spectra of Sr_2_ScO_3_F:Mn and Sr_2_TiO_4_:Mn at room temperature. Sr_2_ScO_3_F:Mn showed deep-red emission owing to transition of Mn^4+^ giving a peak at 697 nm. Obvious two excitation bands in 300–460 nm and in 480–580 nm are attributed to spin-allow ^4^A_2g_→^4^T_1g_ and ^4^A_2g_→^4^T_2g_ transition of Mn^4+^ ions, respectively, while a weak excitation band owing to spin-forbidden ^4^A_2g_→^2^T_2g_ transition is difficult to distinguish and it may be embedded in the tail of the ^4^A_2g_→^4^T_1g_ band as observed in other titanates and tantalates (Sasaki et al., 2014; Wang et al., 2014; Takeda et al., 2015, 2017). Interestingly, the emission from Sr_2_ScO_3_F:Mn was much stronger than that from Sr_2_TiO_4_:Mn; the internal and external quantum yields of Sr_2_ScO_3_F:Mn excited at 345 nm at room temperature (50.5 and 43.5%) were much higher than those of Sr_2_TiO_4_:Mn excited at 380 nm (3.4 and 2.5%). The Mn^4+^-emission from fluoride hosts consists of some very sharp lines while that from oxide hosts is broad (Zhou et al., 2016; Adachi, 2018). The emission from Sr_2_ScO_3_F:Mn is broad as well as Mn^4+^-activated oxide phosphors despite presence of the Sc-F bond. This means that influences of F upon the photoluminescence property of Sr_2_ScO_3_F:Mn are not significant. In Sr_2_ScO_3_F:Mn, it is preferred from the charge compensation that one fluorine is replaced with one oxygen when Mn^4+^ is substituted for Sc^3+^. Such co-substitution results in the formation of MnO_6_ octahedra which give broad Mn^4+^-emission. The spectra of CaSiAlN_3_:Eu, which is the representative red-emitting phosphor activated with Eu^2+^, are also shown in Figure 4. The Sr_2_ScO_3_F:Mn emission is sharper and stronger than the CaSiAlN_3_:Eu emission however the wavelength of the Sr_2_ScO_3_F:Mn emission is excessively long, that is, almost the half portion of emission is located in the invisible region (λ > 700 nm). CaAlSiN_3_:Eu can be excited by blue-LEDs (λ = 450–470 nm) more efficiently than Sr_2_ScO_3_F:Mn while Sr_2_ScO_3_F:Mn can be excited by near ultraviolet LEDs (λ = 350–400 nm) more efficiently than CaAlSiN_3_:Eu.

**Figure 5:**
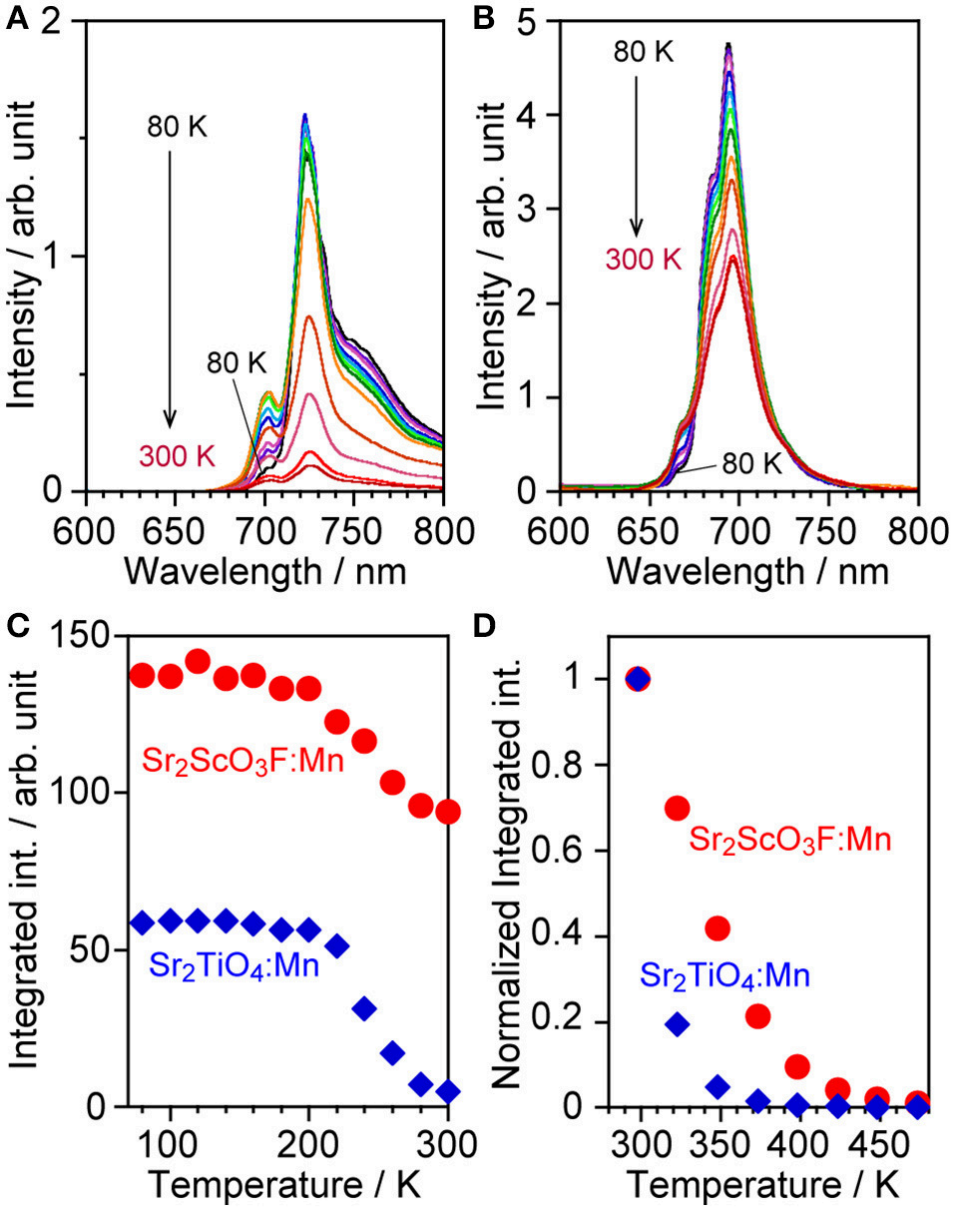
Emission spectra of **(A)** Sr_2_TiO_4_:Mn and **(B)** Sr_2_ScO_3_F:Mn at low temperature (80–300 K), **(C)** relative integrated emission intensity of them, and **(D)** normalized integrated emission intensity of Sr_2_TiO_4_:Mn and Sr_2_ScO_3_F:Mn at high temperature (298–473 K). Excitation wavelengths were 380 and 340–345 nm for Sr_2_TiO_4_:Mn and Sr_2_ScO_3_F:Mn, respectively.

Measurements of thermal quenching were performed at low (80–300 K) and high temperature ranges (298–473 K). Both samples suffered temperature quenching even in the low temperature range especially higher than 200 K as shown in Figures 5A–C. The maximum peak intensity decreased as measurement temperature rose while the emission of anti-Stokes sidebands, which were observed in regions shorter than 710 and 675 nm in Sr_2_TiO_4_:Mn and Sr_2_ScO_3_F:Mn, respectively, was enhanced due to transition of excited electrons to upper vibration states by thermal energy (Wu et al., 2017; Adachi, 2018). It results in the non-obvious decreases in the integrated emission intensity up to 200 K. Sr_2_ScO_3_F:Mn exhibited stronger emission than Sr_2_TiO_4_:Mn at all temperatures, moreover, the intensity of Sr_2_TiO_4_:Mn at 80 K was lower than that of Sr_2_ScO_3_F:Mn at 300 K. Thus, Sr_2_TiO_4_:Mn exhibited more remarkable thermal quenching in comparison with Sr_2_ScO_3_F:Mn. In the high temperature range (298–473 K), significant thermal quenching occurred in both samples as shown in Figure 5D, however Sr_2_ScO_3_F:Mn showed lesser thermal quenching than Sr_2_TiO_4_:Mn. At 373 K, Sr_2_ScO_3_F:Mn showed 20% of emission intensity in comparison with that at 298 K whereas emission from Sr_2_TiO_4_:Mn was completely quenched.

### Band structures of Sr_2_TiO_4_:Mn and Sr_2_ScO_3_F:Mn

**Figure 6:**
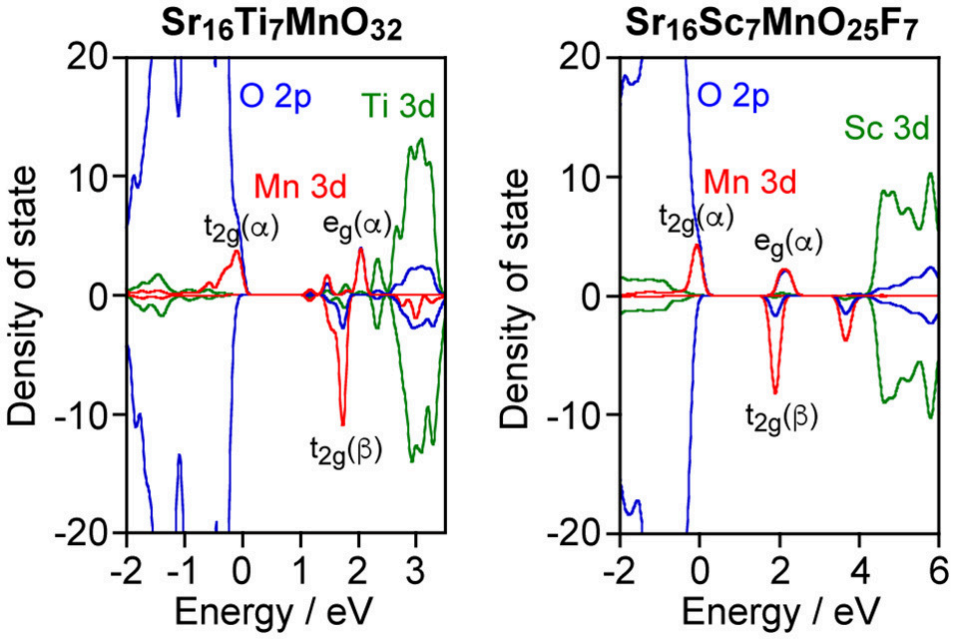
PDOS of Sr_16_Ti_7_MnO_32_ and Sr_16_Sc_7_MnO_25_F_7_.

**Figure 7:**
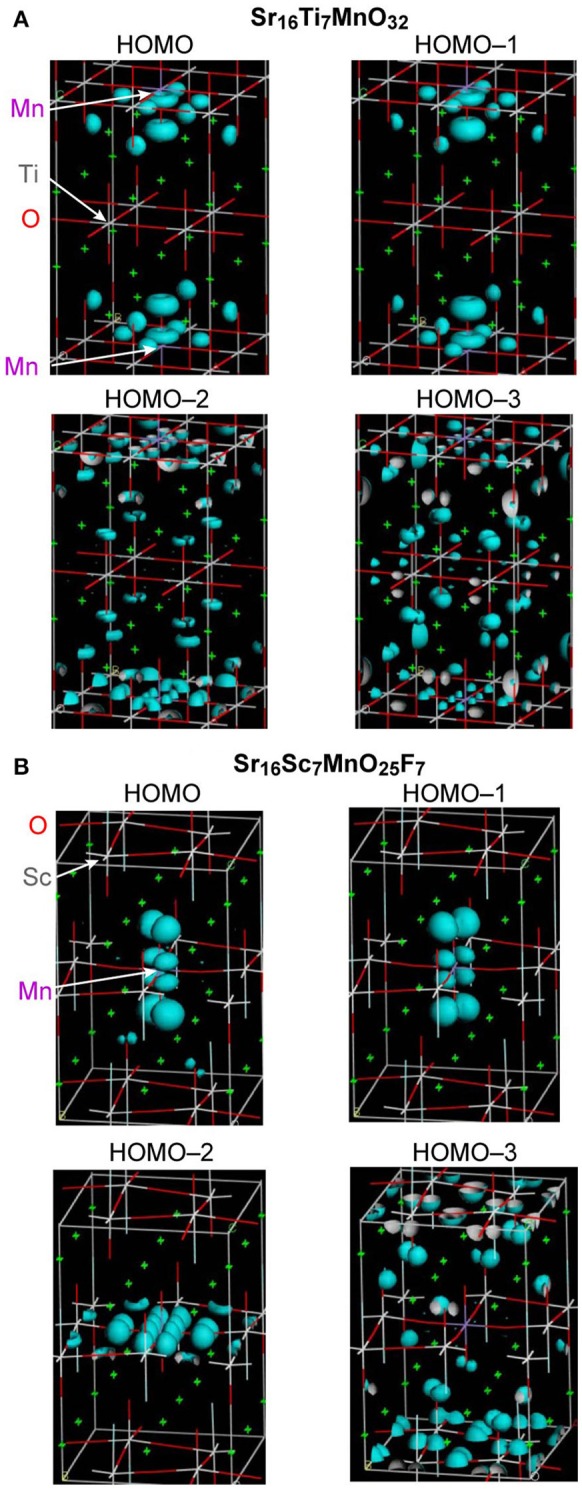
Electron density contour maps of top four occupied orbitals for **(A)** Sr_16_Ti_7_MnO_32_ and **(B)** Sr_16_Sc_7_MnO_25_F_7_.

As described above, Sr_2_ScO_3_F:Mn showed superior characteristics, that is, higher emission intensity and lesser thermal quenching, to Sr_2_TiO_4_:Mn. Relative position of Mn 3d orbitals to the valence and conduction bands of host materials is an important factor for Mn^4+^-activated phosphors as we have reported previously (Takeda et al., 2015, 2017). Therefore, band structures of Sr_2_TiO_4_:Mn and Sr_2_ScO_3_F:Mn were investigated by the DFT method. Figure 6 depicts projected density of states (PDOS) near the band gap of Sr_16_Ti_7_MnO_32_ and Sr_16_Sc_7_MnO_25_F_7_ corresponding to Sr_2_TiO_4_:Mn and Sr_2_ScO_3_F:Mn. In Figure 6, positive and negative values in DOS represent DOS for up-spin (α) and down-spin (β) electrons, respectively, and 0 eV of energy represents the Fermi level. In Sr_2_TiO_4_:Mn, the valence and conduction bands of host are composed of O 2p and Ti 3d orbitals, respectively, like SrTiO_3_:Mn. In PDOS of Sr_2_TiO_4_:Mn, the t_2g_(α) orbitals of Mn look to be located slightly higher position than the valence band however a tail of t_2g_(α) is embedded in the valence band. The DFT calculation reveals that Sr_2_TiO_4_:Mn has absolutely different feature in the relative position of Mn 3d orbitals to the valence band from SrTiO_3_:Mn, in which the t_2g_(α) orbitals are deeply embedded in the valence band (Takeda et al., 2015). Although a part of t_2g_(α) orbitals is located in positive energy region, it doesn't indicate the presence of empty t_2g_(α) orbitals. The total numbers of electrons calculated for both Sr_16_Ti_7_MnO_32_ and Sr_16_Sc_7_MnO_25_F_7_ models were 443. In the quintet state, the numbers of occupied orbitals should be 223 and 220 for α- and β-electrons, respectively. If the top of t_2g_(α) orbital of Mn 3d is empty, the lowest unoccupied molecular orbital for α-electron (#224 α-orbital) should be Mn 3d located near 0 eV. However, #224 α-orbital is not Mn 3d located around 0 eV but Mn3d orbital below the conduction band [indicated as e_g_(α) in Figure 6] in both models. The small portion of the occupied orbitals beyond the Fermi level observed in PDOS is due to broadening of energy widths of orbitals by the smearing treatment in the process of PDOS creation. Thus, it has been confirmed that all Mn 3d orbitals around 0 eV are occupied ones. The PDOS of Sr_2_ScO_3_F:Mn shows that the t_2g_(α) orbitals of Mn is located slightly higher position than the valence band without tailing portion at a lower energy side. The electron density contour maps for top four occupied molecular orbitals including the highest occupied molecular orbital (HOMO) are compared to see details of the differences between Sr_2_TiO_4_:Mn and Sr_2_ScO_3_F:Mn (Figure 7). In Sr_2_ScO_3_F:Mn, contribution of the occupied Mn 3d orbitals is seen only in the top three occupied orbitals (from HOMO to HOMO−2) and the forth highest occupied orbital (HOMO−3) is composed of only O 2p orbital. On the other hand, small contribution of the Mn 3d orbital is also seen in HOMO−3 of Sr_2_TiO_4_:Mn although Mn 3d orbitals mainly contribute to top three occupied orbitals. If hybridization between O 2p and t_2g_(α) of Mn 3d is small, the occupied Mn 3d orbitals appear in only three orbitals. The appearance of Mn 3d in four orbitals in Sr_2_TiO_4_:Mn (from HOMO to HOMO−3) indicates stronger hybridization between O 2p and Mn 3d than Sr_2_ScO_3_F:Mn. It is also noticed in PDOS that energy gap between e_g_(α) of Mn 3d and the bottom of conduction band is larger in Sr_2_ScO_3_F:Mn than Sr_2_TiO_4_:Mn. It reflects the remarkably wider band gap of Sr_2_ScO_3_F than Sr_2_TiO_4_.

**Figure 8:**
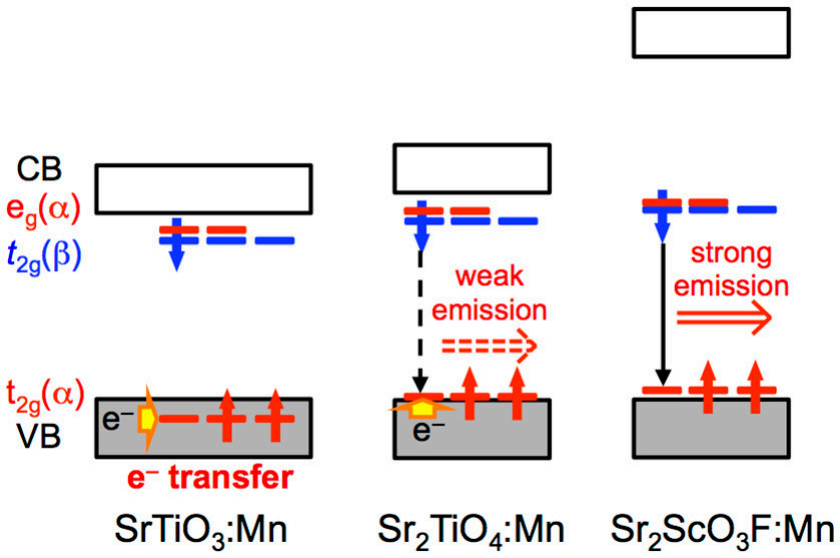
Proposed mechanism for SrTiO_3_:Mn, Sr_2_TiO_4_:Mn, and Sr_2_ScO_3_F:Mn.

Figure 8 illustrates proposed mechanism based on photoluminescence measurements and band structure calculations for Mn^4+^-activated SrTiO_3_, Sr_2_TiO_4_, and Sr_2_ScO_3_F. The most significant difference in photoluminescence property between Sr_2_TiO_4_:Mn and SrTiO_3_:Mn is the appearance of Mn^4+^-emission in Sr_2_TiO_4_:Mn at room temperature. The difference in the relative position of t_2g_(α) orbitals of Mn between SrTiO_3_:Mn and Sr_2_TiO_4_:Mn is of importance in explanation for the appearance of Mn^4+^-emission in Sr_2_TiO_4_:Mn. In SrTiO_3_:Mn, the t_2g_(α) orbitals embedded in the valence band facilitate thermal quenching via the electron transfer from the valence band to the empty t_2g_ in the excited state ^2^E_g_, resulting in low quenching temperature, ~100 K (Takeda et al., 2015). In contrast to SrTiO_3_:Mn, t_2g_(α) in Sr_2_TiO_4_:Mn is located slightly higher position than the valence band. Therefore, Sr_2_TiO_4_:Mn shows Mn^4+^-emission even at room temperature. Interaction between TiO_6_ octahedra may affect the relative position of t_2g_(α) orbitals of Mn. Each TiO_6_ octahedron connects to six TiO_6_ octahedra in SrTiO_3_ while TiO_6_ connects to four TiO_6_ octahedra in the perovskite slab in Sr_2_TiO_4_ as shown in Figure 1, indicating that degree of energy delocalization is higher in three-dimensional SrTiO_3_ than two-dimensional Sr_2_TiO_4_. The smaller interaction between TiO_6_ octahedra may cause less interaction between O 2p and occupied of Mn 3d orbitals, that is, t_2g_(α) orbitals. Thus, the advantage of two-dimensional layered perovskite structure for Mn^4+^-activated phosphors can be explained by the smaller interaction of MO_6_ octahedra. Such discussion can be applied to another Mn^4+^-activated titanate phosphor La_2_MgTiO_6_:Mn with a B-site ordered double perovskite structure, which is an efficient Mn^4+^-activated phosphor with 58.7% of an internal quantum yield (Takeda et al., 2015). In La_2_MgTiO_6_, each TiO_6_ octahedron is surrounded by six MgO_6_ octahedra, meaning that TiO_6_ is isolated from other TiO_6_ octahedra even though the perovskite-type structure (Lee et al., 2000). Thus, the structure of La_2_MgTiO_6_ can be regarded as a quasi-zero-dimensional structure with respect to the connection between TiO_6_ octahedra. The less interaction of TiO_6_ in La_2_MgTiO_6_:Mn leads the larger energy gap between the valence band and t_2g_(α) of Mn, resulting in the superior photoluminescence efficiency to Sr_2_TiO_4_:Mn. In Sr_2_ScO_3_F:Mn, the t_2g_(α) orbitals of Mn 3d are located above the valence band with a slightly larger energy gap than Sr_2_TiO_4_:Mn due to the small hybridization between O 2p and Mn 3d as described above. On the other hand, the larger energy gap between e_g_(α) and the bottom of conduction band in Sr_2_ScO_3_F:Mn suppresses quenching via photoionization, in which an electron in e_g_(α) orbitals in the excited state is transferred to the conduction band and then is relaxed without emission (Takeda et al., 2017). Thus, the two factors, the less interaction between t_2g_(α) and the valence band and the large energy gap between e_g_(α) and the bottom of conduction band, positively affect the smaller thermal quenching in Sr_2_ScO_3_F:Mn than Sr_2_TiO_4_:Mn, resulting in the stronger emission.

## Conclusions

Photoluminescence properties of Mn^4+^-activated strontium titanates, SrTiO_3_:Mn with three-dimensional bulky perovskite structure and Sr_2_TiO_4_:Mn with two-dimensional layered perovskite structure, have been compared in this research. Sr_2_TiO_4_:Mn shows Mn^4+^-emission even at room temperature despite no emission from SrTiO_3_:Mn. In addition, the results in our systematic research suggest that the less interaction between MO_6_ octahedra of B-site cation in the perovskite family provides positive influences in Mn^4+^-emission. Comparison between Sr_2_TiO_4_:Mn and Sr_2_ScO_3_F:Mn indicates that ScO_5_F octahedra are preferable constituents to TiO_6_ ones for the Mn^4+^-activated phosphors. Thus, the present research demonstrates that scandium, which is one of optically inert rare earth elements, is a useful element as a major constituent for design of Mn^4+^-activated phosphors.

## Author contributions

HidK managed all experiments and wrote the manuscript. YT performed experiments on synthesis of samples and evaluation of photoluminescence properties of them. HisK performed band structure calculations. MakK and MasK planned experiments and made discussion about the results.

### Conflict of interest statement

The authors declare that the research was conducted in the absence of any commercial or financial relationships that could be construed as a potential conflict of interest.
